# NUAK1 and NUAK2 Fine-Tune TGF-β Signaling

**DOI:** 10.3390/cancers13133377

**Published:** 2021-07-05

**Authors:** Reinofke A. J. van de Vis, Aristidis Moustakas, Lars P. van der Heide

**Affiliations:** 1 Swammerdam Institute for Life Sciences, University of Amsterdam, Science Park 904, 1098 XH Amsterdam, The Netherlands; r.vandevis@uva.nl; 2 Department of Medical Biochemistry and Microbiology, Science for Life Laboratory, Uppsala University, Box 582, SE-75123 Uppsala, Sweden; aris.moustakas@imbim.uu.se

**Keywords:** NUAK1, NUAK2, AMPK, TGF-β, SMAD, LKB1

## Abstract

**Simple Summary:**

TGF-β is a growth factor implicated in a plethora of processes and malignancies, which include cancer and fibrosis. Via binding to its receptor, TGF-β activates a complex intracellular signal transduction pathway, which is controlled by many forms of positive as well as negative feedback. The integrated sum of this feedback determines the outcome and cellular response to TGF-β. In this review, we discuss the role of NUAK1 and NUAK2, a subgroup of the 5′AMP-activated protein kinase family, in providing feedback on intracellular TGF-β signaling. In addition, we discuss how NUAKs mechanistically augment or attenuate the TGF-β response to steer the cell towards a specific output. Understanding the role of NUAKs may aid in developing specific therapeutic agents to combat TGF-β-dependent disease.

**Abstract:**

Transforming growth factor-β (TGF-β) signaling plays a key role in governing various cellular processes, extending from cell proliferation and apoptosis to differentiation and migration. Due to this extensive involvement in the regulation of cellular function, aberrant TGF-β signaling is frequently implicated in the formation and progression of tumors. Therefore, a full understanding of the mechanisms of TGF-β signaling and its key components will provide valuable insights into how this intricate signaling cascade can shift towards a detrimental course. In this review, we discuss the interplay between TGF-β signaling and the AMP-activated protein kinase (AMPK)-related NUAK kinase family. We highlight the function and regulation of these kinases with focus on the pivotal role NUAK1 and NUAK2 play in regulating TGF-β signaling. Specifically, TGF-β induces the expression of NUAK1 and NUAK2 that regulates TGF-β signaling output in an opposite manner. Besides the focus on the TGF-β pathway, we also present a broader perspective on the expression and signaling interactions of the NUAK kinases to outline the broader functions of these protein kinases.

## 1. TGF-β Signaling, SMADs and Negative Feedback

Transforming growth factor-β (TGF-β) signaling regulates embryonic development and organ or tissue homeostasis during the life-span of all multicellular organisms. This evolutionarily conserved family of cytokines regulates a wide variety of cellular processes, extending from proliferation and apoptosis to differentiation and motility [1]. The large number of cytokine family members and their multifunctional roles explain why TGF-β signaling pathways are implicated in many biological contexts [2]. Consequently, aberrant TGF-β signaling is associated with diverse human diseases, which include cancer, fibrosis and immune diseases [3].

Even though the TGF-β family comprises several related cytokines, for the purpose of this perspective we will focus on the TGF-β subfamily. This subclass consists of three TGF-β proteins, usually referred to as the TGF-β 1, 2 and 3 isoforms for historical reasons [1]. The TGF-β signaling pathway is activated upon dimeric ligand binding to the TGF-β receptor II (TβRII), which will recruit and phosphorylate the TGF-β receptor I (TβRI), forming a heterotetrameric receptor complex [4]. Following this complex formation, the canonical (or small mother against decapentaplegic (SMAD)) signaling pathway can be initiated through the recruitment and phosphorylation of receptor-activated SMADs (R-SMADs), namely SMAD2 and SMAD3. Phosphorylated SMAD2 and SMAD3 dimerize and recruit the common SMAD (co-SMAD) SMAD4, forming a functional SMAD complex able to translocate into the nucleus to regulate gene expression by cooperating with several transcription factors. Besides SMAD-dependent signaling, a broad range of non-canonical (or non-SMAD) pathways can be activated by TGF-β receptor complexes. These include several mitogen-activated protein kinase (MAPK) pathways, consisting of the extracellular signal-related kinase (ERK), c-Jun N-terminal kinase (JNK) and p38 pathways involved in transcriptional regulation. Rho-like GTPase signaling can also be activated by the TGF-β receptors via as yet unknown mechanisms involved in cell adhesion and motility. Another target is the phosphatidylinositol-3-kinase (PI3K/AKT) pathway that operates via mammalian target of rapamycin (mTOR) signaling, which regulates cell survival [5].

TGF-β signaling has multiple biological functions in different cell types and under different biological conditions, which, among other controlling mechanisms, requires a feedback system to regulate signaling quantitatively. These feedback loops are best represented by the inhibitory SMAD (I-SMAD) family, which control TGF-β signaling at several points in the cascade. The I-SMAD family has two members, SMAD6 and SMAD7, with SMAD7 being the specific antagonist for the TGF-β ligands. Upon the TGF-β-mediated transcriptional induction of SMAD7, this I-SMAD is able to bind to TβRI, which hinders SMAD2/SMAD3 phosphorylation and subsequent SMAD2/SMAD3/SMAD4 complex formation, thereby abolishing signal transduction. SMAD7 is also responsible for the proteasomal degradation of both the TGF-β receptor complexes as well as SMAD4 via recruitment of several E3 ligases, such as SMAD ubiquitylation regulatory factor (Smurf) 1, Smurf2, WW Domain-Containing E3 Ubiquitin Protein Ligase 1 (WWP1) or neural precursor cell-expressed developmentally downregulated 4-like (NEDD4-2) [6].

Thus, TGF-β-mediated SMAD signaling transcriptionally induces *SMAD7* gene expression, which then negatively regulates both receptor and SMAD functions. Additional negative feedback mechanisms on TGF-β signal transduction are achieved through the regulation of different TGF-β target genes, governed by the SMAD complex. One example involves the protein kinase death-associated protein-kinase-related 2 (DRAK2). Upon rapid induction of DRAK2 by TGF-β, DRAK2 could associate with the TβRI and obstruct the recruitment of SMAD2/SMAD3 to the TGF-β receptor complex [7]. Another gene target is the serine–threonine kinase salt-inducible kinase (SIK). TGF-β signaling induces the upregulation of SIK, which subsequently could associate and cooperate with both SMAD7 and Smurf2 to downregulate TβRI [8,9]. The above presents a general overview of TGF-β signaling and some of its key molecular components, which will later be used in the discussion of the crosstalk between TGF-β and protein kinases.

## 2. Exploring the NUAK Kinase Family

SIK is a member of the AMP-related family of kinases (ARKs), consisting of 12 kinases that show close similarity in terms of catalytic domain properties with the metabolic sensor protein AMP-activated protein kinase (AMPK) [10]. This family is mainly known for its role in regulating cellular metabolism, with additional functions involved in cellular processes including cell cycle, proliferation and cell polarity [11]. Two relatives of SIK, NUAK family kinase 1 (NUAK1) and NUAK family kinase 2 (NUAK2), were recently characterized to similarly participate in the modulation of TGF-β signaling [12]. The formal names NUAK1 (first described as AMPK-related protein kinase 5, Ark5 [13]) and NUAK2 (first described as sucrose nonfermenting-like kinase, SNARK [14]) stem from the words Nu (novel) and AMP-related kinase (NUAK) subfamily of ARKs. Both NUAKs share homology with the catalytic subunit of AMPK and can be activated on the consensus site in the activation T loop of ARK family kinases [15]. The tumor suppressor liver kinase B1 (LKB1) has been identified as the master upstream regulator of all members of the ARK family, apart from maternal embryonic leucine zipper kinase (MELK), as demonstrated by in vitro phosphorylation assays of recombinant ARK family kinases in the presence of the LKB1/Mo25/STRAD trimeric complex that constitutes the active LKB1 kinase complex [16]. The mutation of the conserved threonine residue of the T-loop in the ARK family kinases abolishes this phosphorylation. Additional in-cell assays using LKB1 knockout cells have demonstrated the dependency of ARK function on the upstream LKB1 kinase activity [16]. This general model of AMPK family activation by LKB1 has been confirmed in mouse skeletal muscle cells for NUAK2 specifically; the cell type-specific knockout of mouse *Lkb1* caused the inactivation of NUAK2 downstream from a stimulus generated by muscle contraction, an effect that physiologically links to the activation of glucose transport into muscle cells, which energetically supports the contractions [17]. Furthermore, analysis of cell adhesion regulation by NUAK1, as explained in detail later, was ablated when *Lkb1* knockout mouse embryonic fibroblasts (MEFs) were assayed [18]. Yet other functions of NUAK1, such as p53 phosphorylation [19] and mitochondrial aggregation in neuronal axons that regulates the branching of the axon at the position of the aggregates [20], have been demonstrated to require the upstream action of the LKB1 complex. Additional activators of AMPK are Ca^2+^/calmodulin-dependent protein kinase II (CAMKII) and TGF-β-activated kinase 1 (TAK1) [15]. Similar to AMPK, both NUAK1 and NUAK2 could be activated by Ca^2+^ signaling. However, instead of CAMKII, protein kinase C ⍺ (PKC⍺) was identified as the kinase activating NUAK1. The possibility of CAMKII involvement in NUAK2 activation was excluded, but the responsible Ca^2+^-dependent kinase has not been identified yet [21]. TAK1 involvement in NUAK kinase activation remains elusive. 

Human NUAK1 was first described as a mediator of AKT (or protein kinase B; PKB) signaling and found to promote cell survival under nutrient deprivation by regulating p53 activity [13]. The human *NUAK1* gene is located on chromosome 12 and consists of seven exons. It encodes a 661-amino acid-long protein with a predicted molecular weight of 76 kDa. The highly homologous mouse *Nuak1* gene is located on chromosome 10 and consists of eight exons and is 658 amino acids long. Direct phosphorylation by AKT on an alternative site other than LKB1 was shown to activate NUAK1 kinase activity, making NUAK1 the only ARK member activated by AKT [13]. Nuclear dbf2-related kinase 2 (NDR2) activated NUAK1 upon insulin-like growth factor (IGF) stimulation [22]. The physiological roles of NUAK1 show contrasting functions. The regulation of the mitotic director Polo-like kinase 1 (PLK1) by NUAK1 stimulated the S-phase progression of the cell cycle [23]. Contrarily, NUAK1 has been shown to control senescence and ploidy by stabilizing the genomic stability regulator large tumor suppressor kinase 1 (LATS1) [24]. NUAK1 was also found to directly modulate p53 activity in an LKB1-dependent manner, resulting in cyclin-dependent kinase inhibitor p21^WAF1^-induced cell cycle arrest [19]. These reports illustrate the complex and context-dependent functions of NUAK1 with both pro-survival (cell cycle stimulatory) and tumor suppressive (cell cycle inhibitory) properties.

NUAK2 was originally identified upon transcriptional induction after UV-B radiation exposure of keratinocytes [14]. The human *NUAK2* gene is located on chromosome 1 and consists of eight exons. It encodes a 628-amino acid-long protein with a predicted molecular size of 70 kDa. The highly homologous mouse *Nuak2* gene is located on chromosome 1 and encodes for two transcript variants. The longest A isoform has eight exons and is 639 amino acids long, whereas the shorter B isoform has seven exons and is 631 amino acids long. The shorter isoform lacks an in-frame exon in the coding region. Interestingly, this lacking exon codes for the amino acid sequence GRSRLVTV, which contains a putative PKB/AKT phosphorylation site (RxRxxT, where x represents any amino acid). However, there are no data to suggest that this is a functional phosphorylated motif. The human NUAK2 is also devoid of this sequence and as such is slightly more similar to the mouse B isoform. Similar to its relatives, NUAK2 is activated by several cellular stresses, including increased AMP or decreased ATP levels, glucose or glutamine deprivation, oxidative or endoplasmic reticulum stress and DNA damage in a cell type-specific manner [25]. NUAK2 has been observed to protect skeletal myocytes against stress-induced apoptosis by mediating the pro-survival Rho signaling pathway [26]. 

To date, the regulation of the expression of the NUAK kinase family has not been clearly represented in the literature. Two distinct human tissue RNA-sequencing datasets, either from the Genotype–Tissue Expression (https://gtexportal.org/home/ accessed on 24 June 2021) project or as published by Fagerberg et al., 2014 [27], showed comparable expression patterns for both kinases in many tissues. The gene expression datasets of both NUAK kinases can be observed in the GTEx browser: for NUAK1, https://gtexportal.org/home/gene/NUAK1, and for NUAK2, https://gtexportal.org/home/gene/NUAK2, accessed on 24 June 2021, respectively. NUAK1 exhibits a ubiquitous expression pattern in human tissue, with particularly high expression in the brain, arteries, skin, adipose tissue and several parts of the female reproductive system. Low expression levels of NUAK1 are found in blood, spleen, pancreas, as well as gastrointestinal tract components. Unlike its relative, NUAK2 shows a more selective tissue expression pattern. Intriguingly, NUAK1 and NUAK2 show opposing expression patterns in several tissues. NUAK2 is highly expressed in various gastrointestinal tract tissues, especially in esophageal mucosa. Other examples of high NUAK2 expression include the kidney and spleen as well as a great representation in whole blood. NUAK2 can also be found in the cerebellar part of the brain, accompanying NUAK1. Coinciding expressions of the two NUAK family members can also be found in skin, lung and vaginal tissue. 

In a pathological context, NUAK function has frequently been associated with tumor development and progression, exhibiting both tumor-suppressive and oncogenic characteristics. The cell cycle inhibitory functions of NUAK1 were discussed previously [19,24]. Additional examples of pro-oncogenic functions include the AKT-dependent NUAK1 activity, which was found to exacerbate both colorectal and pancreatic tumor malignancy, as illustrated by the increased tumor growth and invasiveness induced upon NUAK1-mediated synthesis of matrix metalloproteinases (MMP) [28]. NUAK1 was also found to inhibit caspase-dependent apoptosis through the inactivation of caspase-6 in colorectal cancer cells [29]. Furthermore, when NUAK1 was activated by PKC, it protected MYC-driven tumor cells from cell death [21]. Similarly, NUAK2 was found to protect cells from apoptosis after being induced by the death receptor cluster of differentiation 95 (CD95) and tumor necrosis factor ⍺ (TNF⍺). NUAK2 expression also increased the motility and invasive character of breast cancer cells [30]. NUAK2 is associated with melanoma tumor growth, both by controlling cell cycle progression via cyclin-dependent kinase 2 (CDK2), as well as by regulating migration [31,32].

## 3. Opposing Roles of NUAK1 and NUAK2 in TGF-β Signaling 

**Figure 1:**
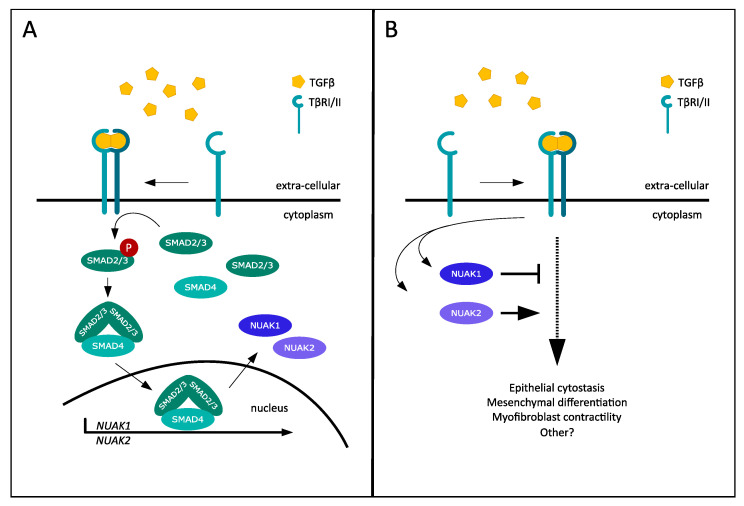
TGF-β-mediated induction of the NUAK kinases and their opposing effects on TGF-β signaling output. (**A**) TGF-β ligand binding to its respective receptor leads to the activation of SMAD2/SMAD3, as well as subsequent SMAD2/SMAD3/SMAD4 complex formation and nuclear translocation, and ultimately results in the transcriptional induction of NUAK1 and NUAK2. (**B**) Upon their induction, the opposing effects of the NUAK kinases on the TGF-β pathway are depicted, with NUAK1 inhibiting and NUAK2 enforcing various established TGF-β signal outputs.

Novel roles for both NUAK1 and NUAK2 in the context of TGF-β signaling have recently been identified, exposing an intricate TGF-β feedback mechanism with opposing functions for these two kinases [12]. TGF-β transcriptionally induced both NUAK1 and NUAK2 in various mammalian cell types, including mouse breast epithelial cells, human breast cancer cells and human immortalized keratinocytes, as well as primary human foreskin fibroblasts, in a TβRI- and SMAD4-dependent manner (Figure 1A). Additionally, ERK1/2 and p38 pathway inactivation diminished the TGF-β-dependent induction of NUAK2 in mouse epithelial cells, which suggests an additional role for MAPK signaling in NUAK2 production. The identification of an intronic enhancer region in the *NUAK2* gene, which promotes NUAK2 transcription upon SMAD2/3 complex binding, uncovered the mechanism of TGF-β-mediated inducibility [12]. SMAD3 engaged in NUAK2-mediated signaling besides the mechanism of transcriptional induction, as was observed by the ability of NUAK2 to associate with the linker and MH2 domains of SMAD3. The knockdown of NUAK2 resulted in the downregulation of SMAD3, suggesting that association with NUAK2 increases SMAD3 stability, which supports TGF-β signal transduction [12]. Similarly, complex formation between NUAK2 and TβRI was also observed. Thus, NUAK2 is able to associate with two essential signaling components of the TGF-β pathway. Furthermore, the induction of several TGF-β target genes in fibroblasts, including the extracellular matrix genes *FIBRONECTIN 1* (encodes FN1), *SERPINE1* (encodes plasminogen activator inhibitor 1, PAI1), and *TIMP1* (encodes tissue inhibitor of metalloproteinase-1, TIMP1), diminished in the absence of NUAK2. This effect was perpetuated at the level of protein expression, where NUAK2 silencing reduced the expression of FN1 in epithelial cells. This affirms the positive modulation NUAK2 exerts on the transcriptional output of TGF-β. Intriguingly, the knockdown of NUAK1 resulted in increased expression of FN1 in HaCaT human keratinocytes, suggesting an opposite role for NUAK1 [12].

One characteristic response of TGF-β signaling is the induction of cell cycle arrest in epithelial cells [2]. Silencing of NUAK1 in HaCaT keratinocytes caused a significant increase in the number of growth-arrested cells, illustrating the negative effect of NUAK1 on this specific TGF-β-mediated response [12]. The effect of NUAK2 was examined with the NMuMG-Fucci cell model to investigate cell cycle dynamics [33]. NUAK2 knockdown resulted in the exact opposite response, with a significant reduction in growth arrest observed. Upon TGF-β stimulation, the cytostatic response induced in epithelial cells is required for the subsequent process of epithelial to mesenchymal transition (EMT). TGF-β is a well-established potent promoter of EMT in epithelial tissue [2]. Both at the level of transcriptional programming and cell physiology, NUAK2 silencing prevented epithelial cell transition to a mesenchymal phenotype [12].

Mesenchymal differentiation cell models further confirmed the dual role of NUAK kinases in regulating TGF-β signaling. Another TGF-β-mediated response is to drive fibroblast–myofibroblast differentiation [34]. In fibroblasts, this response is facilitated by the upregulation of several cytoskeletal proteins, including ⍺-smooth muscle actin (⍺SMA) and calponin, differentiating towards specialized contractile myofibroblasts [35]. Protein expression of ⍺SMA and calponin in fibroblasts increased in the absence of NUAK1, whereas the silencing of NUAK2 decreased the inducibility of these TGF-β target genes. Investigating the contraction ability of differentiating myofibroblasts in the absence of either NUAK kinase showed that NUAK1 knockdown enhanced gel contractility, indicating increased TGF-β mediated differentiation in the absence of NUAK1. The opposite effect was observed after NUAK2 knockdown, by decreased gel contractility [12]. Additionally, TGF-β signaling is involved in the arrest of myoblast–myotube differentiation. This myogenic differentiation is halted by TGF-β through the suppression of myosin heavy chain (MHC) and myogenin induction [36]. This suppressive response of TGF-β intensified in the absence of NUAK1 in mouse myoblasts, whereas NUAK2 silencing caused an upregulation of both MHC and myogenin expression, promoting myogenic differentiation [12]. 

These findings identify a novel feedback mechanism for the TGF-β pathway, with central roles for the TGF-β-induced NUAK kinase family. This feedback is generated when TGF-β induces the expression of NUAK1 and NUAK2 (Figure 1A), with the subsequent action of NUAK1 as a negative contributor (negative feedback) and NUAK2 as a positive enforcer (positive feedforward) of TGF-β signal output (Figure 1B). The fact that TGF-β regulates NUAK1 and NUAK2 expression in cells of diverse origin, and the evidence that NUAK1 and NUAK2 regulate TGF-β signaling in both epithelial cells and fibroblasts, suggest that the crosstalk between TGF-β and NUAK kinases may be widespread and cell type-independent. However, mechanisms that regulate acto-myosin contraction are most probably relevant to mesenchymal cell types, whereas mechanisms involved in cell cycle arrest may be more relevant to epithelial cells. One aspect worth studying deeper is the specific involvement of NUAK family isoforms in the regulation of TGF-β signaling. So far it remains unclear as to whether TGF-β induces all isoforms of the NUAKs and if the specific isoforms function according to the above model. The fact that the NUAK kinase family is able to steer the outcome of multiple physiological roles of TGF-β illustrates the general integrated function of the NUAK family in fine-tuning TGF-β signaling. 

## 4. NUAKs at the Crossroads of TGF-β and LKB1 Signaling

The inducibility of the NUAK family by TGF-β and the subsequent signal modulation of these kinases expose a novel means of cross-talk between the AMPK family and the TGF-β pathway. However, both NUAK kinases require upstream activation for proper functioning. Thus, in order to have a full understanding of the mechanism by which the interplay between the ARKs and the TGF-β pathway occurs, an analysis of the roles and mechanisms of NUAK kinases via LKB1, CAMKII, AKT, NDR2 and possible other upstream signaling proteins is necessary.

The tumor suppressor kinase LKB1 is the main upstream regulator known to activate both NUAK1 and NUAK2 [16]. Intriguingly, direct interplay between LKB1 and TGF-β signaling has been described as well. LKB1 could associate with SMAD4, mediated by the scaffolding protein LKB1-interacting protein 1 (LIP1). This results in the phosphorylation of SMAD4 by LKB1, thereby inhibiting the transcriptional activity of SMAD4 [37]. The tumor-suppressive function of LKB1 is illustrated by studies performed with LKB1-deficient mesenchymal cells, where loss of LKB1 showed decreased TGF-β ligand production as well as deficient paracrine TGF-β signaling. This resulted in increased epithelial proliferation and hamartomatous polyp development [38]. The association of LKB1 with the TGF-β pathway and its ability to modulate signal output suggests the possibility of the upstream involvement of LKB1 in NUAK activation. Furthermore, studies using MEFs from Lkb1 knockout mice showed reduced production of ⍺SMA stress fibers and decreased contractility, which demonstrates a similar phenotype to the NUAK2-deficient mouse fibroblasts observed by Kolliopoulos et al. (2019) [12,39]. The drive to myofibroblast differentiation could be rescued upon exogenous TGF-β stimulation, implying that the mitigation of TGF-β signaling causes impairment in the myofibroblast differentiation process [39]. The resemblance in phenotypes between LKB1- and NUAK-deficient cell studies implies a role for LKB1 in the NUAK-mediated modulation of TGF-β signaling, and highlights the relevance of investigating the involvement of LKB1 in NUAK-dependent signaling.

Identifying the responsible activating kinase(s) will contribute to our understanding of NUAK kinase functioning. Correspondingly, investigating the expression levels and activity of both kinases upon upstream activation combined with the specific conditions required for pathway activation could further disclose the mechanism behind the opposing effects observed by NUAK1 and NUAK2. This will be facilitated by focused studies under different and specific biological contexts, where the mechanisms of NUAK activation may depend on different molecular components. The regulatory effects of NUAK kinases on signaling by additional members of the TGF-β family will be interesting to explore. 

## 5. Additional Dynamics of NUAK Kinase Function

**Figure 2:**
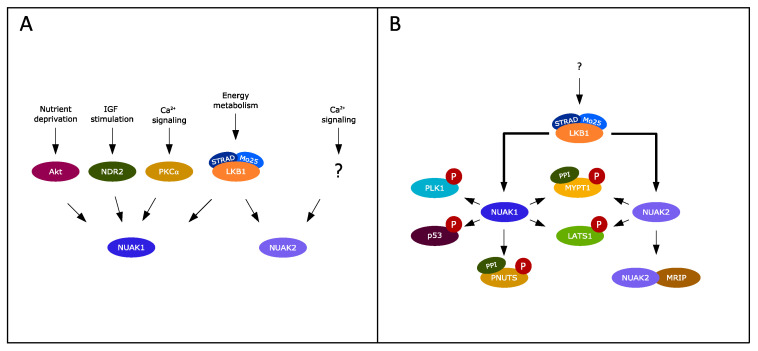
Interactors of NUAK1 and NUAK2. Currently known activators (**A**) and substrates (**B**) of the NUAK kinase family are illustrated. The arrow with a question mark depicts the unidentified kinase downstream of Ca^2+^ signaling for NUAK2 activation in panel A and depicts the upstream kinase activation implied by the LKB1–STRAD–Mo25 complex in panel B.

Besides their role as TGF-β signal modulators, the NUAK family has been implicated in additional cellular processes, including cell proliferation and motility. Their targets and functions have always been closely associated (Figure 2). NUAK1 and NUAK2 are both involved in the regulation of smooth muscle cell contraction through the association and phosphorylation of myosin phosphatase target subunit 1 (MYPT1), the regulatory subunit of protein phosphatase 1 (PP1). Phosphorylation causes the inhibition of the phosphatase activity of PP1^MYPT1^, resulting in the phosphorylation of myosin regulatory light chain (MLC2), thereby stabilizing actin stress fibers and promoting cell detachment [18,40]. Both NUAK kinases are suggested to utilize a similar function in this context. NUAK1 specifically has been linked to the regulation of the cell cycle via the inhibition of PP1^MYPT1^. The mitotic director PLK1 plays a key role in this mechanism, as it can be inhibited by the PP1^MYPT1^ complex. Banerjee et al. (2014) reported the NUAK1-mediated activation of PLK1 via PP1^MYPT1^ inhibition, linking NUAK1 function to the cell cycle [23]. Furthermore, PLK1 mediates the degradation of NUAK1 by the Skp, Culling and F-box^βTrCP^ E3 ligase complex (SCF^βTrCP^), which correlates to the reciprocal expression of PLK1 and NUAK1 in the G2-M versus the S-phase of the cell cycle [23]. The phosphorylation of MYPT1 by NUAK1 can be mediated by ROS, and results in the nuclear import of the antioxidant regulator NF-E2-related factor (NRF2) via inhibition of the protein kinase glycogen synthase kinase 3β (GSK3β) that keeps NRF2 in the cytoplasm [41]. NUAK1 was found to regulate the activity of the spliceosome via interaction with the PPI^PNUTS^ subunit, another isoform of the PP1 phosphatase [42]. Besides the direct phosphorylation of MYPT1, NUAK2 has also been found to modulate actin filaments in a kinase-independent way through association with the myosin phosphatase Rho-interacting protein (MRIP). This complex formation leads to the dissociation of MRIP from MYPT1, thereby indirectly facilitating actin stress fiber formation [40].

Hippo is another fundamental cellular pathway where NUAK family members were recently characterized as central mediators. The Hippo pathway controls the transcriptional programming governing stemness, mobility, differentiation and cell fate, with the transcriptional co-activators yes-associated protein (YAP) and transcriptional co-activator with PDZ-binding motif (TAZ) as the major downstream effectors [43]. In the absence of upstream inhibitory signaling by LATS1/2 and mammalian Ste20-like kinase 1 and 2 (MST1/2), YAP/TAZ promote target gene expression. Dysregulation of the Hippo pathway is frequently associated with tumorigenesis and malignancy [43]. NUAK2 was transcriptionally induced after direct YAP/TAZ activation and was found to promote YAP/TAZ signaling through a positive feedback loop. 

Subsequently, NUAK2 phosphorylated LATS1, thereby alleviating the inhibition of YAP/TAZ and reinforcing YAP/TAZ activity [44]. This positive feedback loop was augmented by MYPT1-mediated cytoskeletal modulation. Pathologically, the NUAK2 activation of YAP/TAZ promoted tumor growth and liver overgrowth [45]. YAP-driven NUAK2 expression was found to play a significant role in squamous cell carcinoma development [46]. NUAK1 has also been shown to be able to phosphorylate LATS1 [24], but whether NUAK1 exerts a similar or opposing function to NUAK2 on Hippo pathway signaling remains elusive. 

Recently, NUAK2 and the Hippo system have been shown to be of high relevance to a crucial stage in development of the human central nervous system: namely, the closing of the neural tube during embryogenesis [47]. Defects in closing the neural tube lead to a developmental brain defect called anencephaly: the absence of a large portion of the brain. In a family with repetitive cases of anencephaly, exome sequencing of two fetuses with anencephaly and their parents identified a recessive germline mutation (22 base pair deletion and 1 base pair insertion) in the *NUAK2* gene [47]. This deletion resulted in a kinase-deficient NUAK2 protein, failure to phosphorylate downstream targets such as LATS proteins, and consequent cytoplasmic YAP retention. The authors suggested that NUAK2 activity is required for control of the Hippo pathway during embryonic development and cytoskeletal rearrangements during neuronal tube closure. Interestingly, fibroblasts from the anencephalic fetus that were reprogrammed into induced pluripotent stem cells (iPSCs) and differentiated into neural progenitor cells (NPCs) resulted in a dramatic decrease in NUAK1 levels, whereas NUAK2 levels remained unaltered. This suggests that NUAK1 levels depend on NUAK2 kinase activity for normal expression levels, which adds an additional layer of complexity to NUAK signaling [47]. The already established crosstalk between TGF-β/SMAD and Hippo/YAP/TAZ signaling in various biological contexts, including embryonic development [48,49], suggest interesting future avenues wherein such crosstalk could be analyzed by examining the integration of these two major developmental pathways at the level of NUAK family kinases. An overview of the currently known mechanisms of NUAK signaling is provided for NUAK1 (Table 1) and NUAK2 (Table 2).

## 6. Selective NUAK Inhibitors

As described above, NUAK1 and NUAK2 have diverse functions and can behave in a context-dependent manner. As NUAKs could present possible therapeutic targets in various malignancies, it is vital to target NUAK1 or NUAK2 specifically to minimize off-target effects. One possible way to target NUAKs is by inhibiting their kinase activity with small molecule kinase inhibitors or peptide decoys. Inhibitors can be directed at the catalytic site, which would be an approach for a pan-NUAK inhibitor. However, the similarity between the kinase domain of NUAK1 and NUAK2 as well as other kinases would possibly limit specificity. Exploiting the differences between NUAK1 and NUAK2 would allow the generation of more selective inhibitors. NUAK inhibitors have been described that inhibit either both NUAKs or are NUAK-specific. The inhibitors WZ4003 and HTH-01-015 belong to the first generation of NUAK inhibitors and can be considered relatively specific, as they inhibited the NUAKs without inhibiting a panel of 139 other kinases [50]. Both inhibitors target NUAK1, whereas HTH-01-015 has at least a 1000-fold lower affinity for NUAK2. Both WZ4003 and HTH-01-015 are presumed to act as ATP-competitive inhibitors, but their precise mechanism of action is unknown. Over the last few years additional NUAK inhibitors have been described [51], but to date a specific NUAK2 inhibitor has not been generated. Peptide decoys could possibly be utilized to maximize specificity. Additionally, modified sequences from specific NUAK1 or NUAK2 downstream targets could be used to selectively inhibit an NUAK isoform and cut off a NUAK signaling branch. 

## 7. Conclusions and Perspectives

The identification of several substrates has aided in our understanding of the scope of NUAK signaling as regulators of various fundamental cellular processes. The susceptibility for tumorigenesis and malignancy upon dysregulation of NUAK signaling illustrates the drive to identify ways by which we can experimentally preserve cellular balance with NUAK1 and NUAK2. Furthermore, their variation in different physiological outcomes indicates a context-dependent specificity that drives kinase function. The identification of opposing roles for NUAK1 and NUAK2, which create a functional balance in TGF-β signaling output, exposes a complex signaling mechanism that can possibly apply to additional signaling pathways. Furthermore, this places the NUAK kinase family at the center of TGF-β signaling, creating an important role for the NUAKs in a wide range of TGF-β-mediated cellular processes. Despite this, the NUAK family remains a relatively poorly understood subclass compared to the other ARK members. Further studies are necessary to gain a complete understanding of the mechanism and function of the NUAK kinases, in both the normal and pathological context, in order to develop specific therapeutic interventions and inhibitors that target NUAK signaling, as this can benefit patients suffering from diverse diseases, including fibrotic, degenerative and immune diseases, and cancer.

## Figures and Tables

**Table 1:** NUAK1 kinase signaling. Currently identified upstream signaling, and activators and targets of NUAK1 and its downstream effects on cellular phenotype are illustrated.

NUAK1					
Model	Upstream Signaling	Direct Activator	Target Substrate	Cellular Phenotype	Ref
HepG2 cells	Glucose starvation	Akt	ATM	Stimulated p53-mediated cell survival during glucose starvation	[13]
PANC-1 and DLD-1 cell lines	IGF-1 signaling	Akt		Increased MT1-MMP production and subsequent MMP-2 and MMP-9 activation, stimulated tumor invasion and metastasis	[28]
SW480, DLD-1, HCT-15, HCT-116 and WiDr cell lines	IGF-1 signaling	Akt	Caspase-6	Promoted resistance to the extrinsic Fas/FasL—mediated cell death in colorectal cancer cells	[29]
HCT-166, DLD-1 and SW480 cells	IGF-1 signaling	NDR2		Promoted tumor cell survival and invasion	[22]
WI-38 cells, HEK293		LKB1	LATS1	Increased aneuploidy and induced senescence	[24]
HEK293, *Lkb1^−/−^* and *Nuak1^−/−^*MEFs	Cell detachment	LKB1	PP1 subunit MYPT1	Promoted myosin II-mediated cell detachment	[18]
A549 and Hep3B cells	Glucose starvation	LKB1	p53	Promoted p21/WAF1 induced cell cycle arrest	[19]
Mouse cortical neurons (*Lkb1* KO or *Nuak1* KO)		LKB1		Stimulated presynaptic mitochondria immobilization and cortical axon branching	[20]
U2OS cells		CDK and subsequent PLK	E3 ligase SCF^β^^TrCP^ binding	SCF^β^^TrCP^-mediated degradation of Nuak1. Controlled proliferation by stimulating S-phase and PLK1-mediated mitosis	[23]
HeLa cells	Ca^2+^ signaling	PKC⍺	Raptor	Inhibited MTORC1-regulated cell growth	[21]
U2OS, SW480 and HCT116 cell lines	Oxidative stress		PP1 subunit MYPT1	Promoted colorectal cancer formation by suppressing GSK3β-dependent inhibition of NRF2 nuclear mobilization	[41]
NMuMG, HaCaT, HEK293T, AG1523 cell lines	Expression induced by TGF-β			Inhibited TGF-β-mediated epithelial cytostasis, mesenchymal differentiation and myofibroblast contractility	[12]
U2OS cells			PP1 subunit PNUTS	Promoted spliceosome activity	[42]

**Table 2:** NUAK2 kinase signaling. Currently identified upstream signaling, and activators and targets of NUAK2 and its downstream effects on cellular phenotype are illustrated.

NUAK2					
Model	Upstream Signaling	Direct Activator	Target Substrate	Cellular Phenotype	Ref
BHK fibroblasts, NRKC cells	Elevated AMP concentration (AICAR), glucose deprivation	Observed auto-phosphorylation	SAMS peptide		[14]
MCF7(-FB), HEK293T, ACHN cell lines	Expression induced by Fas receptor activation, requiring NF-κB signaling			Protected tumor cells against Fas-induced apoptosis and promoted motility and invasion	[30]
BHK, HEK293, INS-1, H4IIE, NRKC cell lines	Diverse cellular stresses			Cell type-specific kinase activity regulated upon nutrient starvation, cellular ATP decrease and/or AMP increase, ER stress, osmotic stress, oxidative stress or UV-B radiation	[25]
HEK293 cells			LATS1		[24]
Mouse skeletal muscle	In situ and in vitro muscle contraction, treadmill activity	LKB1	*Suggested* AS160 and TBC1D1	Stimulated contraction, stimulated glucose transport in muscle	[17]
U2OS and HeLa cell lines	Induced expression upon growth signals and actin stress fiber alterations		MRIP (kinase-independent association)	MLCP inhibition and promoted actin stress fibers	[40]
C32, SM2-1 and mel18 melanoma cell lines	*PTEN*-deficiency			CDK2 activation promoted melanoma tumor growth	[31]
C2C12 myoblasts and mouse skeletal muscle	Increased expression with muscle differentiation and metabolic stress		MYPT1	Promoted Rho kinase signaling-mediated myocyte survival during stress	[26]
MDA-MB231 cells, HEK293T	Induced expression by serum-activated YAP/TAZ signaling		LATS1/2	Positive feedforward reinforcement of YAP/TAZ signaling, increased cell proliferation	[44]
HeLa cells	Ca^2+^ signaling				[21]
HuCCT-1, H69 and SNU475 cell lines	YAP-mediated induction		MYPT1	Actomyosin-regulated YAP amplification, promoted YAP driven liver cancer cell proliferation	[45]
NMuMG, HaCaT, HEK293T, AG1523	Expression induced by TGF-β			Stimulated TGF-β-mediated epithelial cytostasis, mesenchymal differentiation and myofibroblast contractility	[12]
Various types of skin tumors				Close association between NUAK2 and YAP expression in squamous cell carcinoma and Bowen’s disease	[46]
Patient derived NPCs	Loss-of-function NUAK2 germline mutation		LATS2	Hippo signaling regulated neural tube closure through the apical actomyosin network	[47]
